# NLRP3 inflammasome is expressed and regulated in human islets

**DOI:** 10.1038/s41419-018-0764-x

**Published:** 2018-06-25

**Authors:** Fanny Lebreton, Ekaterine Berishvili, Géraldine Parnaud, Caroline Rouget, Domenico Bosco, Thierry Berney, Vanessa Lavallard

**Affiliations:** Cell Isolation and Transplantation Center, Department of Surgery, Geneva University, Hospitals and University of Geneva, Geneva, Switzerland

## Abstract

NRLP3 inflammasome is a protein complex involved in the maturation of IL1β. In the onset of type 1 diabetes as well as in islet transplantation, IL-1β is one of the cytokines involved in the recruitment of immune cells in islets and eventually in islet destruction. Whether IL-1β is produced by islet cells is still under debate and NLRP3 inflammasome-dependent IL-1β production has not been yet determined in human islets. The aim of this study was to determine the expression and the regulation of the NRLP3 inflammasome in human islets. Human islets were stimulated with LPS and successively with ATP (LPS + ATP) in the presence or absence of the inflammasome inhibitor glyburide. Islets were also incubated in hypoxic or normoxic conditions for 24 h in the presence or absence of glyburide. Then, *IL1B* and *NLRP3* expression was studied by real time PCR, protein expression by western blot, protein localization by immunofluorescence and protein secretion by ELISA. LPS + ATP increased gene expression of *NRLP3* and *IL1B*. Glyburide partially prevented this effect. IL-1β protein was localized in β and non-β cells. Moreover, LPS + ATP increased IL-1β protein expression and production, which were prevented by glyburide. Hypoxia increased gene expression of *NRLP3* and *IL1B* and induced IL-1β and caspase-1 production. Finally, hypoxia-induced cell death which was not prevented by inhibition of NLRP3 inflammasome. NRLP3 inflammasome is expressed and plays a role in IL-1β production by human islets. By contrast, NRLP3 inflammasome activation is not involved in islet cell death induced by hypoxia.

## Introduction

Inflammasomes are multi-protein complexes activated in response to infections, inflammations and in autoimmune processes. The best characterized inflammasome is composed of a NOD-like receptor family, pyrin domain containing 3 (NLRP3), an apoptosis-associated speck (ASC)-like protein and pro-caspase 1, and is called NLRP3 inflammasome. The main function of NLRP3 inflammasome is the maturation and the production of interleukin-1 beta (IL-1β). To this end, two distinct signals are involved. The first (priming) signal induces transcription and traduction of pro-IL-1β and NLRP3 via a Toll-like receptor- nuclear factor-kappa B (TLR-NF-κB) pathway. The second signal (activation) results in the NLRP3 inflammasome formation and activation of caspase-1. Activated caspase-1 will then cleave the inactive pro-IL-1β into the active IL-1β form, promoting its secretion^1^. IL-1β is a mediator of inflammation and immune responses playing a central role in the host defence against many pathogens. However, IL-1β overproduction leads to a chronic inflammation and contributes to the pathogenesis of many inflammatory and autoimmune diseases^2^. In type 1 diabetes (T1D), it participates to the recruitment of immune cells in islets, alters insulin secretion and induces β-cell apoptosis^3,4^. IL-1β also plays a pivotal role in cell destruction that occurs after islet transplantation. Studies have shown that an increased production of IL-1β, TNFα, and IFNγ after islet transplantation induces activation of macrophages, then a local production of IL-1β leading to dysfunction and destruction of the graft^5–8^. In solid organ graft, inflammasomes have been shown to play an important role in acute and chronic rejection^9^. However, their expression and role in grafted islets have not been determined. Furthermore, inflammasome-dependent IL-1β production in transplanted islets may be induced by hypoxia. Indeed, islet isolation procedure disrupts vascularization, and even if after transplantation, the process of neovascularization is quickly activated^10^, the restoration of a functional and partial blood flow lasts for weeks^11^ or months for a complete functional vascularization to occur^12^. Hypoxia has been shown to be linked to oxydative stress, process responsible for reactive oxygen species (ROS) generation^13^. Interestingly, studies have shown that ROS are activators of inflammasome and subsequently IL-1β production^14,15^. However, hypoxia as an enhancer of inflammasome has not been yet demonstrated in islets.

A better understanding of mechanisms involved in islet destruction by IL-1β is primordial to try to improve islet viability in T1D and after islet transplantation. Therefore, in this study, we assessed whether NLRP3 inflammasome is expressed and regulated in human islets in physiological and pathophysiological (hypoxia) conditions.

## Materials and methods

### Human islet isolation and culture

Human islets were isolated from brain-dead multiorgan donors using a method previously described^16,17^. Islets were used for research only if not suitable for transplantation. The use of human islets for research was approved by our local institutional ethical committee. After purification, islets were transferred in CMRL 1066 medium containing 5.6 mmol/l glucose and supplemented with penicillin, streptomycin, glutamin, HEPES, and 10% FCS (hereafter referred to as complete CMRL). In all preparations, islets were incubated first at 37 °C for 18–24 h and subsequently at 24 °C for maximum 48 h when required, before to be used in experiments.

### Human islet and islet cells treatments

For immunostaining, human islets were dissociated into single cells as described^18^. After incubation overnight at 37 °C in complete CMRL, 50,000 cells were attached on 35-mm-diameter Petri dishes coated with 0.1 mg/ml of poly-L-lysine and incubated overnight at 37 °C.

Aliquots of 1000 human islets and 50’000 islet cells were incubated 1 h in complete CMRL, in the presence or absence of 200 μM glyburide, used here as an inflammasome inhibitor (Santa Cruz biotechnology, Dallas, Texas, USA). Then, human islets and islet cells were exposed or not to 1 μg/ml LPS (Sigma, Saint-Louis, Missouri, USA) for 4 h or to 5 mM ATP (Sigma, Saint-Louis, Missouri, USA) for 30 min or to 1 μg/ml LPS for 4 h and successively to 5 mM ATP for 30 min (LPS + ATP).

In hypoxia experiments, 1000 human islets were incubated in complete CMRL in hypoxia chamber (1% O_2_) (Stemcell Technologies, Grenoble, France) or in normoxia conditions (21% O_2_) at 37 °C for 24 h in presence or absence of 200 μM glyburide.

### Real-time quantitative PCR analysis

RNA was extracted from human islets using the RNeasy minikit (Qiagen, Courtaboeuf, France). RNA was reverse transcribed using the High Capacity cDNA Reverse transcription kit (ThermoFischer Scientific, Waltham, MA, USA). Gene amplification was achieved with the RT-PCR method using the TaqMan Fast Advance Master Mix (ThermoFischer Scientific). Primers used for amplification were purchased from ThermoFischer Scientific: human Rplp0 (Hs9999902_m1), human IL-1β (Hs01555410-m1), human NLRP3 (Hs00918082-m1). Gene expressions values were normalized to the value of the housekeeping gene RPLP0 and calculated based on the comparative cycle threshold Ct method (2^−ΔCt^ method).

### Immunoblot analysis

Islets were solubilized in lysis buffer (20 mM Tris, 1 mM EDTA, 1 mM EGTA, 150 mM NaCl, 0.5% Triton X-100 and proteases inhibitors) at 4 °C for 10 min. Proteins were separated by SDS-PAGE using a 10% or 15% resolving gel and transferred to a polyvinylidene difluoride membrane (Merck Millipore). The membrane was blocked with saline buffer (10 mm Tris, pH 7.4, 320 mm NaCl) containing 5% bovine serum albumin (BSA) for 1 h at room temperature and blotted overnight at 4 °C with the following antibodies at the dilution indicated by the manufacturer’s instructions: anti-IL-1β (sc:7884, Santa Cruz), anti-HIF (04-1006, Merck Millipore), anti-actin (MAB1501R, Merck Millipore). An ECL protein detection kit (Amersham Biosciences, Piscataway, USA) and a Molecular Omager ChemiDoc XRS+System (Bio-Rad, Hercules, CA, USA) were used for visualization of the bands.

### Immunofluorescence staining

Islet cells, attached on Petri dishes as described above, were fixed in 10% methanol-free formalin, permeabilized with 0.1% Triton X-100 in phosphate buffered saline (PBS), rinsed, incubated in 0.5% BSA in PBS, and then exposed for 2 h to a combination of primary antibodies: a rabbit anti-IL-1β (1:50, Santa Cruz, sc:7884), a guinea pig anti-insulin (1:100, DakoCytomation, Carpinteria, CA, USA), a mouse anti-glucagon (1:4000, Sigma-Aldrich, St Louis, MO, USA) or a rat anti-somatostatin (1:100, Abcam, Cambridge, UK). After rinsing in PBS, islet cells were exposed for 1 h to an adequate combination of fluorescence-labeled secondary antibodies (Jackson ImmunoResearch Laboratories, Rheinfelden, Switzerland). Islet cells were rinsed in PBS and coverslipped before being observed and photographed using a confocal laser scanning microscope, LSM510 META (Zeiss, Feldbach, Germany). Images acquired from the confocal microscope were analyzed for pixel intensity using Image J software. Pixel intensity was quantified separately in manually selected regions of the cells. A region was drawn around each cluster of cells to be measured and the same size region was drawn in an area without fluorescent objects to be used for background subtraction. The net integrated intensity for each cluster of cells was measured by Image J software. To calculate the CTCF (Corrected Total Cell Fluorescence) we used the following formula: CTCF = Integrated density−(Area of selected cell × Mean fluorescence of background readings). In each cluster of cells the number of nuclei were counted and CTCF was normalized to the number of nuclei control.

### Release of IL-1β, caspase-1, and VEGF

When required, islet culture supernatants were collected and stored at −20 °C until use. The amount of IL-1β, caspase-1, and VEGF proteins in the culture medium was measured using human-specific enzyme-linked immunosorbent assay (ELISA) kits (R&D systems, Minneapolis, USA) following the manufacturer’s instructions.

### Analysis of cell death

Cell death was evaluated measuring intact CK18 and cleaved-CK18 fragment using M65^®^ ELISA and M30 Apoptosense^®^ ELISA kits (PEVIVA, TecoMedical, Switzerland). Intact CK18 is released by necrotic cells. CK18 is also cleaved by caspases during apoptosis generating soluble protein fragments. The M65^®^ ELISA kit detects all the forms of CK18 and measures cell death due to both apoptosis and necrosis. Samples react with a solid phase capture antibody M6 directed against CK18 and the HRP (horseradish peroxidase) conjugated M5 antibody directed against a different epitope of CK18. The M30 Apoptosense^®^ ELISA detects caspases-generated CK18 fragment and measures only apoptosis. Samples react with a solid phase capture antibody M5 directed against CK18 and the HRP-(horseradish peroxidase) conjugated M30 antibody directed against the CK18Asp396 neo-epitope. The difference between the values of M65 and the values of M30 quantifies necrosis. The values were normalized to the total protein concentration in the lysate.

### Statistical analysis

Differences between means were assessed either by the Student’s *t* test or by 1-way ANOVA. Where ANOVA was applied, Tukey post hoc analysis was used to identify significant differences between groups.

## Results

### NLRP3 inflammasome is expressed and regulated in human islets

**Fig. 1:**
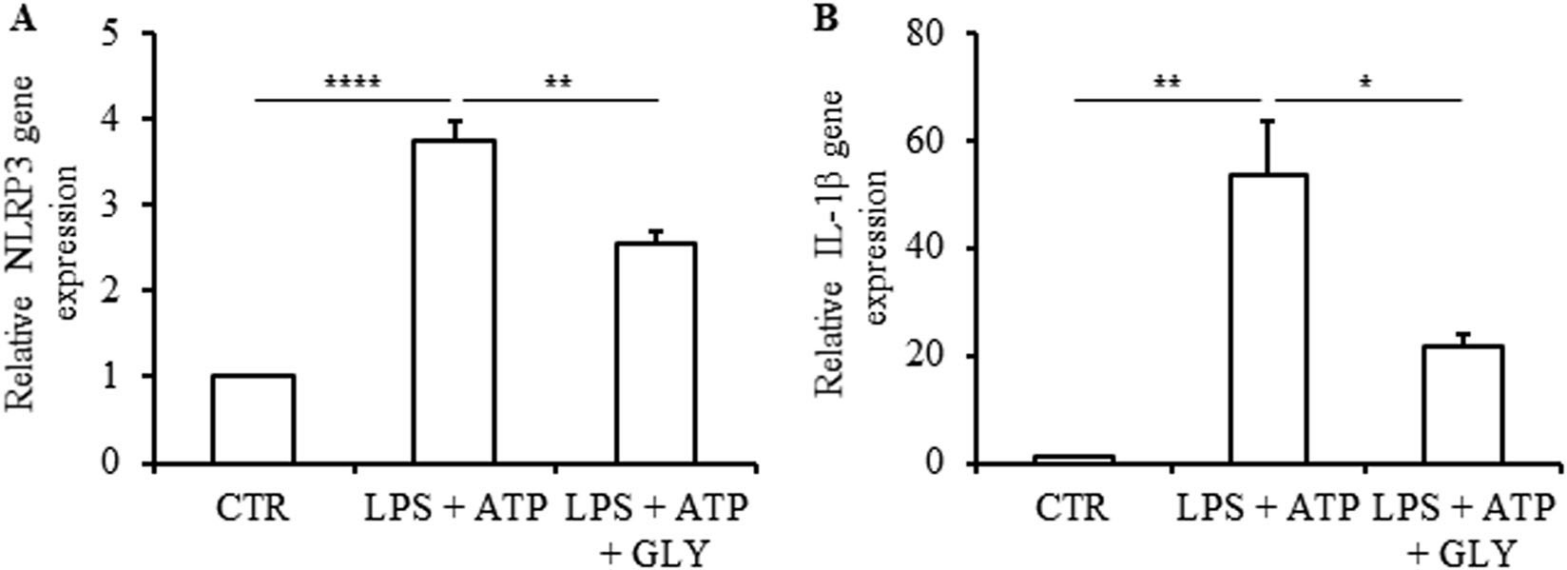
NLRP3 and IL-1β gene expression in human islets in response to inflammasome activation. Human islets were stimulated or not (control, CTR) with 1 μg/ml LPS for 4 h and successively with 5 mM ATP for 30 min (LPS + ATP) in the presence or absence of 200 μM glyburide (GLY). *NLRP3* (**a**) and *IL1B* (**b**) in human islets were quantified by qRT-PCR and expressed relative to CTR. Data are means ± SEM from four experiments (**a**) and three experiments (**b**). *p<0.05, **p<0.01, ****p<0.0001

**Fig. 2:**
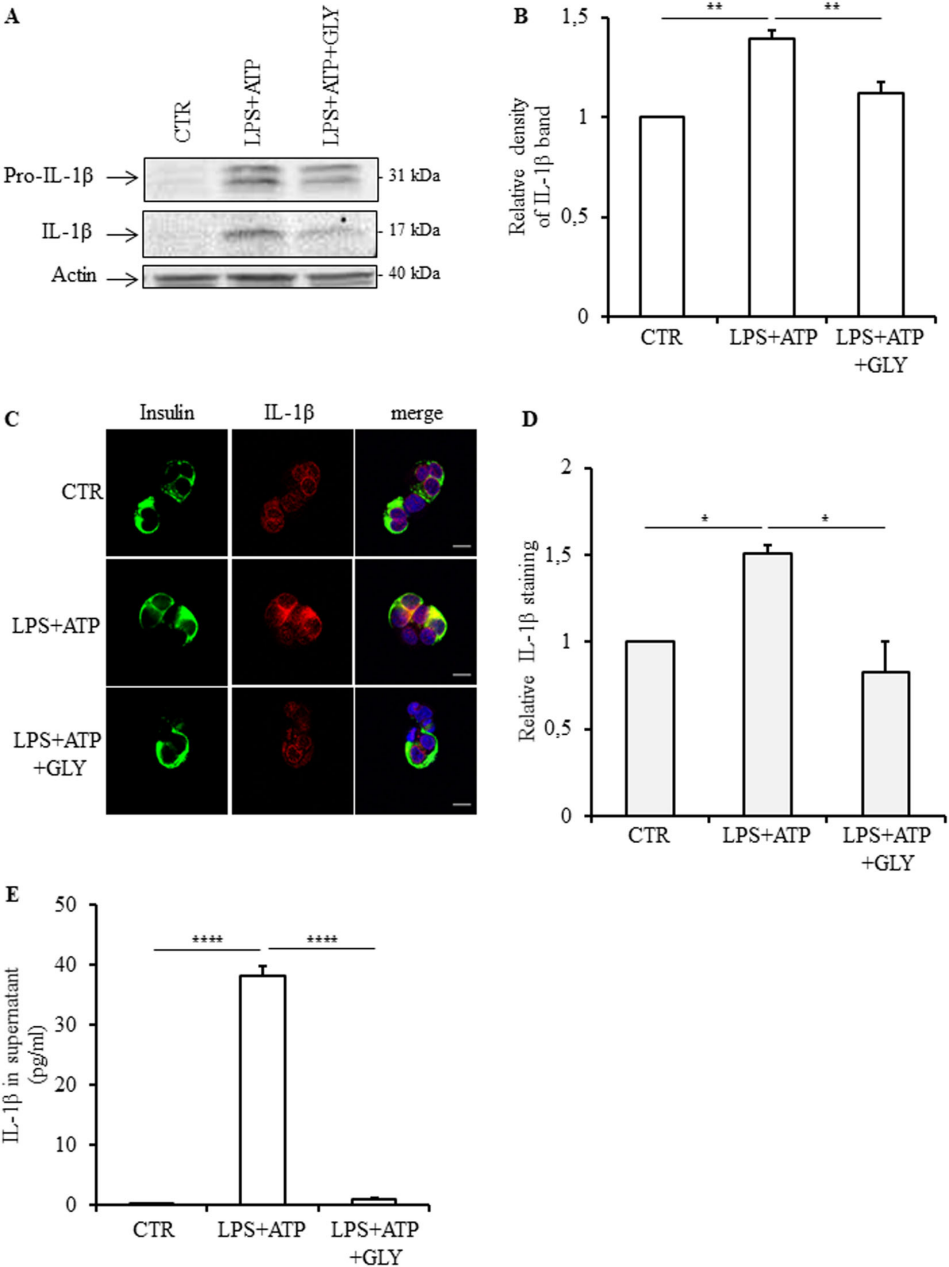
Content and release of IL-1β in human islets in response to inflammasome activation. Human islets were stimulated or not (control, CTR) with 1 μg/ml LPS for 4 h and successively with 5 mM ATP for 30 min (LPS + ATP) in the presence or absence of 200 μM glyburide (GLY). Protein levels of pro- and mature IL-1β forms were analyzed by Western blot (**a**) and density of IL-1β bands quantified (**b**). IL-1β cell localization was analyzed by immunofluorescence (**c**). The red staining (corresponding to IL-1β) was quantified by morphometry and expressed relative to CTR; a total of 79, 88, and 63 cells from three experiments were analyzed for CTR, LPS + ATP and LPS + ATP + GLY, respectively. IL-1β released in the supernatant was quantified by ELISA (**e**). Data are means ± SEM from three (**b** and **d**) and five experiments (**e**). *p<0.05, **p<0.01, ****p<0.0001

Human islets were incubated with LPS for 4 h and successively with ATP for 30 min (LPS + ATP), and *NRLP3* and *IL1B* expressions analysed by real-time quantitative PCR. In response to this treatment, *NRLP3* increased 3.4 ± 1.0 fold (Fig. 1a) and *IL1B* increased 50.4 ± 1.9 fold (Fig. 1b). The NLRP3 inflammasome inhibitor glyburide partially prevented the increased expression of *NLRP3* (Fig. 1a) and *IL1B* (Fig. 1b) induced by LPS and ATP. Under similar conditions, the IL-1β content was analyzed by western blot, its cell localization by immunofluorescence, and its release in culture supernatant by ELISA. As compared to control, LPS + ATP treatment significantly increased IL-1β content and this effect was partially prevented by glyburide (Fig. 2a, b). Double-immunofluorecence labelling for IL-1β and insulin indicated that IL-1β was present in β-cells. Staining for IL-1β was higher in β-cells treated with LPS + ATP as compared to control. This higher staining was attenuated when cells were treated with glyburide (Fig. 2c, d). IL-1β was also present in non-β-cells (Fig. 2c). By multi-immunofluorescence staining, we showed that α-cells (cells labeled for glucagon) and not δ-cells (cells labeled for somatostatin) expressed IL-1β in addition to β-cells (Figure S1A-B). Interestingly, staining for IL-1β in non-β-cells did not increase after treatment with LPS + ATP. Significant amount of IL-1β (38.1 ± 1.8 pg/ml) was found in the supernatant of islets incubated with LPS + ATP, whereas IL-1β was undetectable in the control or present at low level (0.92 ± 0.28 pg/ml) in the presence of glyburide (Fig. 2e). LPS and ATP alone had a significant effect on *IL1B* (Figure S2B) similarly to what observed with LPS + ATP. By contrast, neither LPS nor ATP had an effect on IL-1β release (Figure S2C). Both LPS and ATP had a tendency to increase *NLRP3* (Figure S3A). Taken together, these results show that NLRP3 inflammasome is expressed and regulated in human islets.

### Hypoxia activates NLRP3 inflammasome in human islets

**Fig. 3:**
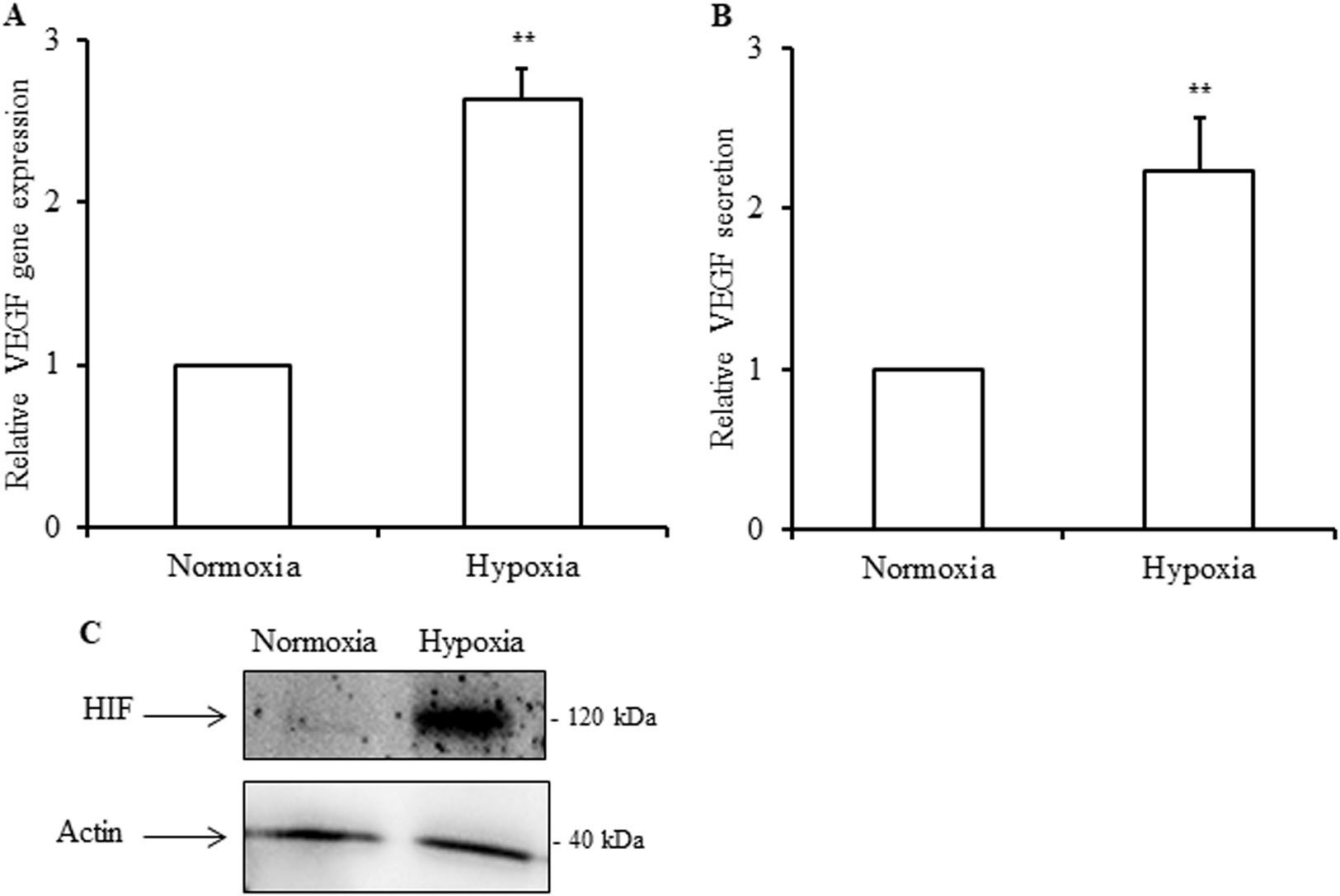
Human islets respond to hypoxia. Human islets were incubated either in control condition (Normoxia) or in 1% O_2_ hypoxia chamber (Hypoxia) for 24 h, and *VEGF* expression (**a**), VEGF released (**b**) and HIF cell content (**c**) analyzed. **a** *VEGF* was evaluated by qRT-PCR. **b** Secreted VEGF was quantified by ELISA. **c** Protein level of HIF was performed by Western blot. Data are means ± SEM from three (**a**) and six experiments (**b**). **p<0.01

**Fig. 4:**
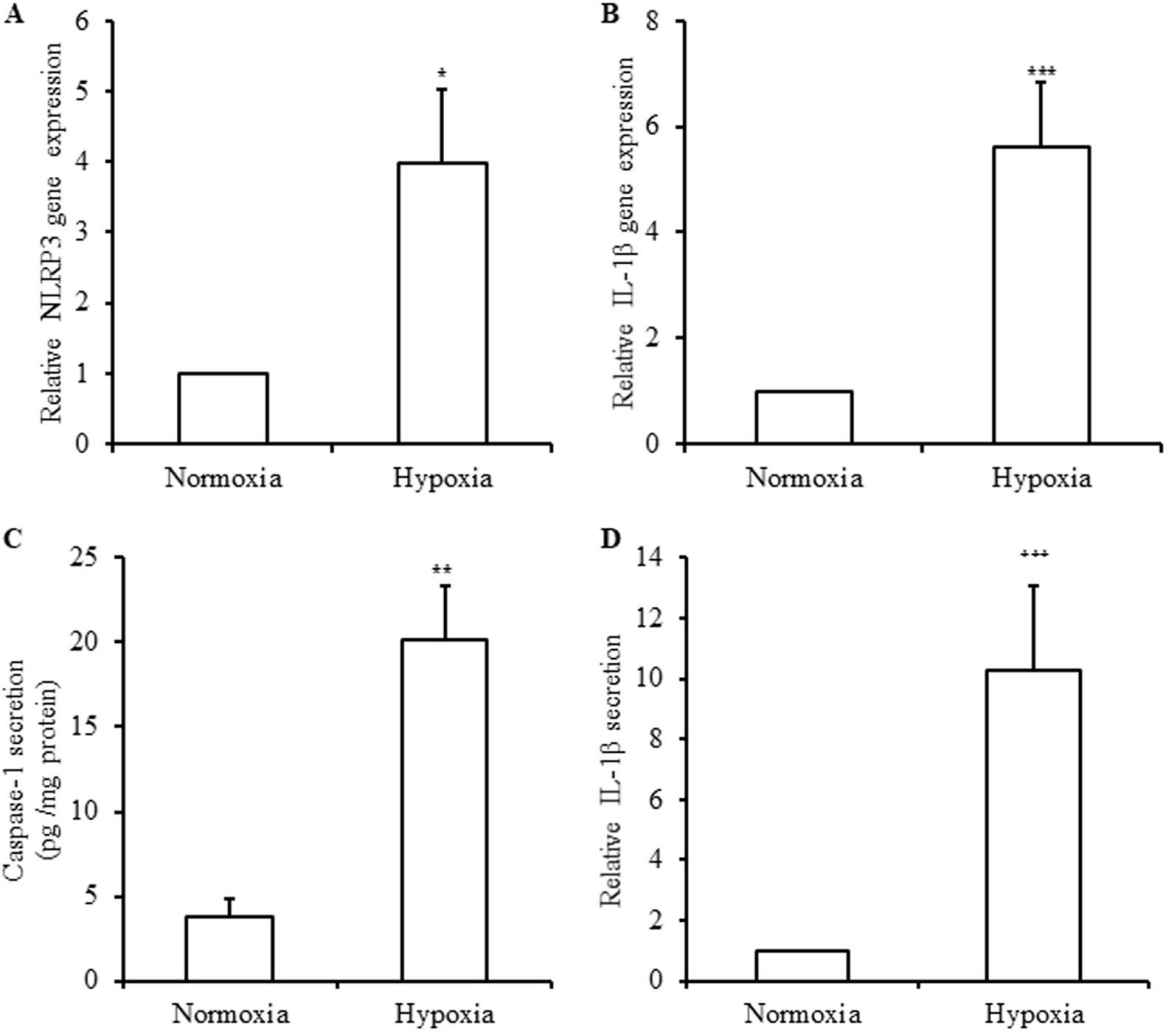
Activation of NLRP3 inflammasome by hypoxia in human islets. Human islets were incubated in control condition (Normoxia) or in 1% O_2_ hypoxia chamber (Hypoxia) for 24 h. *NLRP3* (**a**) and *IL1B* (**b**) were quantified by qRT-PCR and expressed relative to normoxia. Caspase-1 (**c**) and IL-1β (**d**) released were quantified by ELISA. Data are means ± SEM from 6 (**a**), 9 (**b**), 4 (**c**) and four experiments (**d**). *p<0.05, **p<0.01, ***p<0.001

**Fig. 5:**
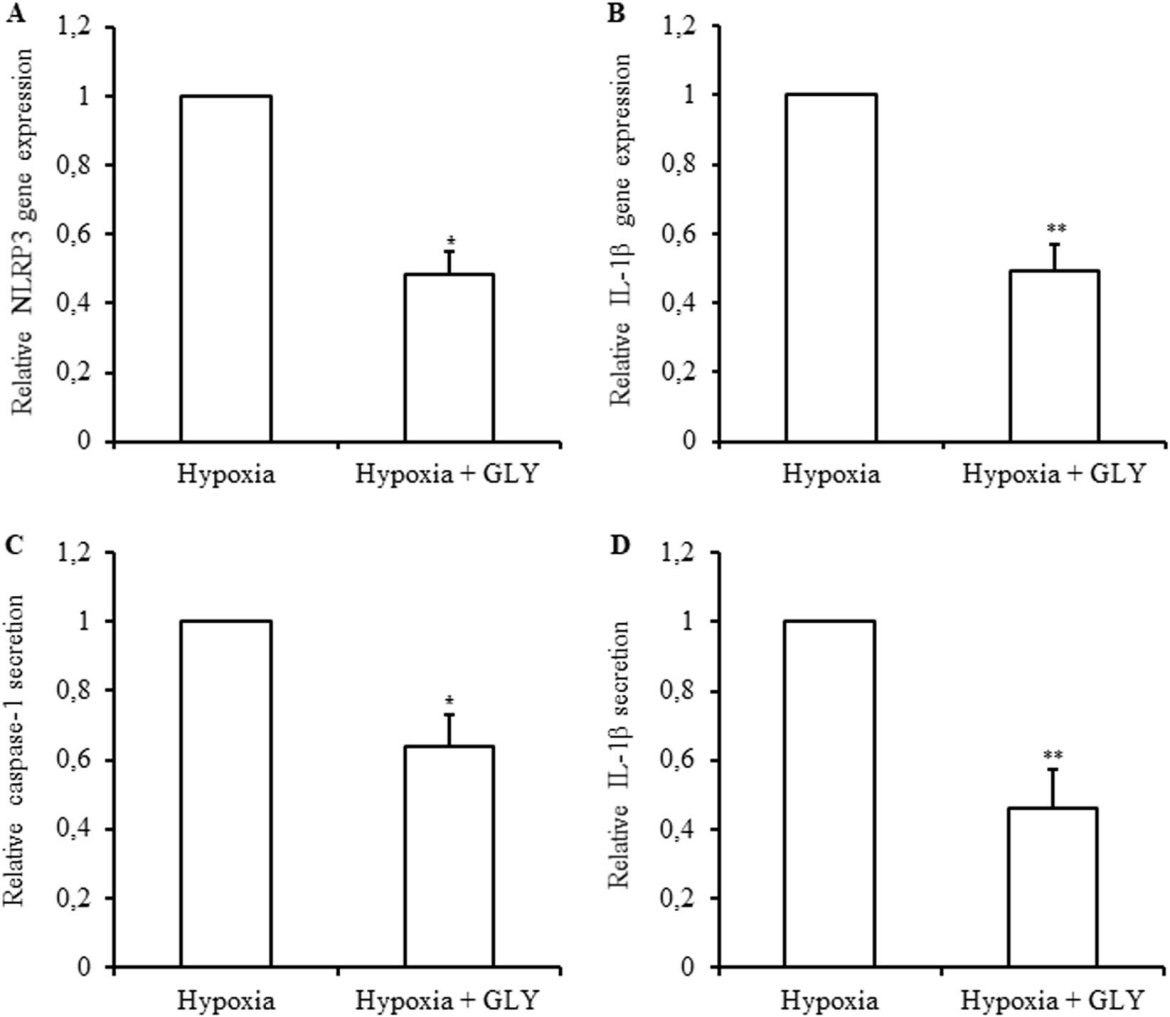
Glyburide prevents activation of NLRP3 inflammasome induced by hypoxia in human islets. Human islets were incubated in 1% O_2_ hypoxia chamber for 24 h in the absence (Hypoxia) or presence or of 200 μM glyburide (Hypoxia + GLY). *NLRP3* (**a**) and *IL1B* (**b**) were quantified by qRT-PCR and expressed relative to Hypoxia. Caspase-1 (**c**) and IL-1β (**d**) released were quantified by ELISA and results expressed relative to Hypoxia. Data are means ± SEM from three experiments. *p<0.05, **p<0.01

Isolated human islets were incubated under normoxic (control, 21% O_2_) and hypoxic conditions (1% O_2_) for 24 h. As expected, Vascular Endothelial Growth Factor (*VEGF)* gene expression (Fig. 3a), VEGF released into the supernatant (Fig. 3b), and Hypoxia-inducible-factor (HIF) protein expression (Fig. 3c) were increased in response to hypoxia as compared to normoxia. Regarding the inflammasome genes, *NRLP3* and *IL1B* expressions were increased 3.6 ± 0.97 and 5.5 ± 1.1 fold, respectively, in response to hypoxia (Fig. 4a, b). Furthermore, the amounts of caspase-1 and IL-1β found in the supernatant of islets incubated under hypoxia were higher as compared to control (Fig. 4c, d). Glyburide partially prevented the increase in *NRLP3* and *IL1B* expressions and IL-1β and caspase-1 secretion induced by hypoxia (Fig. 5). These results indicate that NLRP3 inflammasome is induced and regulated in human islets in response to hypoxia.

### Inhibition of NLRP3 inflammasome does not relieve islet cell death induced by hypoxia

**Fig. 6:**
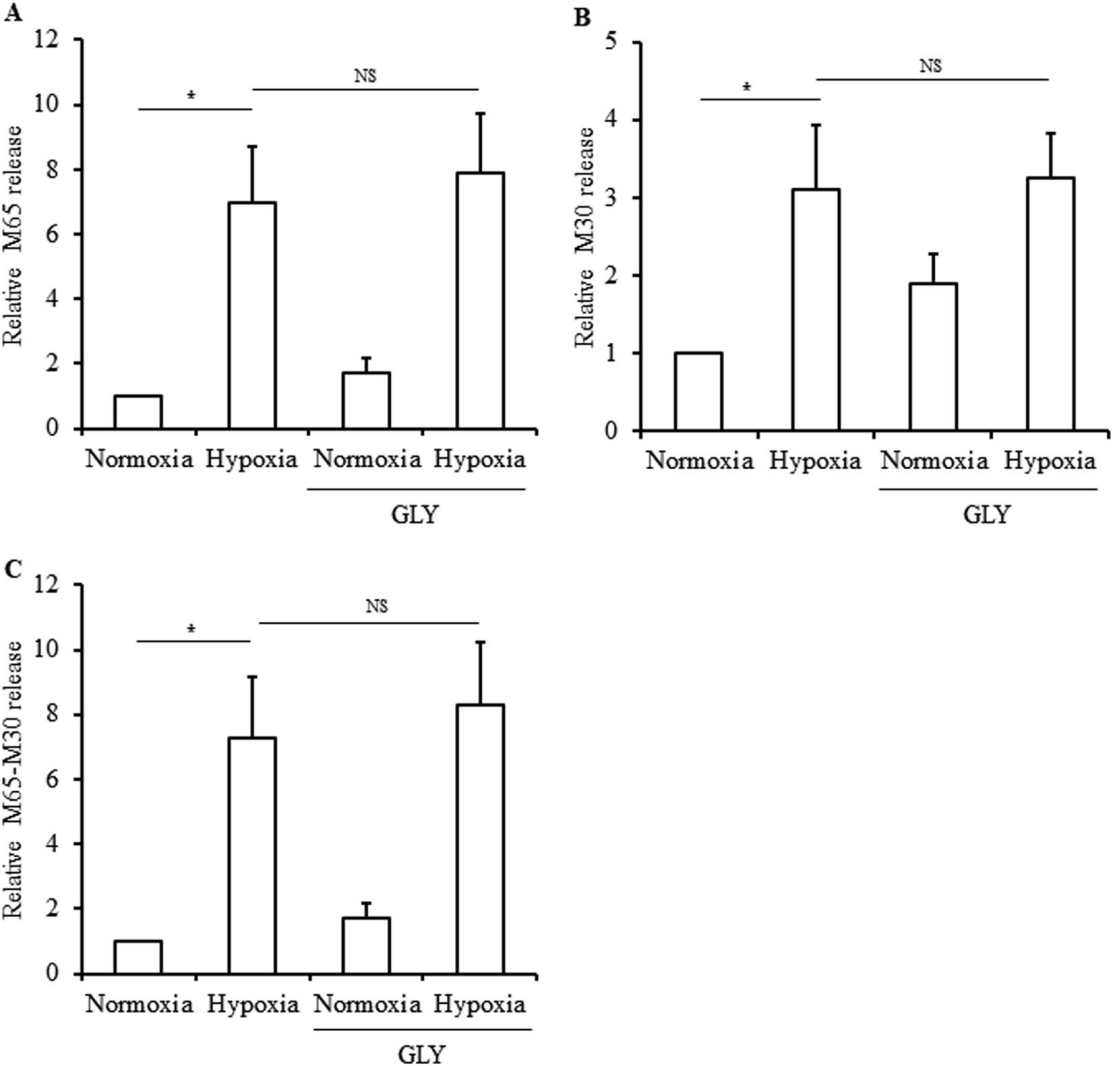
Role of NLRP3 inflammasome in hypoxia-induced cell death in human islets. Human islets were incubated in control condition (Normoxia) or in 1% O_2_ hypoxia chamber (Hypoxia) for 24 h in the presence or absence of 200 μM glyburide (GLY). Cell death was evaluated by Apoptosense^®^ ELISA kits. M65 values express both apoptosis and necrosis (**a**). M30 values express apoptosis (**b**). M65-M30 values (difference between the M65 and M30 values) express necrosis (**c**). NS no significant. Data are means ± SEM from seven experiments. *p<0.05

To determine whether NLRP3 inflammasome is involved in islet cell death induced by hypoxia, NLRP3 inflammasome was inhibited by glyburide under hypoxia and cell death analyzed. We analyzed total cell death, necrosis and apoptosis, by quantifying released intact cytokeratin-18 (CK18) and caspases-generated CK18 fragments. As expected, we observed that hypoxiainduced total cell death (Fig. 6a) comprising apoptosis (Fig. 6b) and necrosis (Fig. 6c). However, inhibition of NLRP3 inflammasome by glyburide did not prevent islet cell death, neither necrosis nor apoptosis. These results indicate that NLRP3 inflammasome is not involved in islet cell death induced by hypoxia.

## Discussion

NLRP3 inflammasome is expressed by innate immune cells, mainly macrophages, and its activation requires two distinct signals. The first signal is mediated by the TLR-NF-κB pathway and results in an increase of the cellular contents of the pro-IL-1β and NLRP3 inflammasome. ATP initiates the second signal, promoting the assemblage and the activation of NLRP3 inflammasome, and leading to the maturation and secretion of IL-1β^19^. In this study, we have investigated the expression and regulation of NLRP3 inflammasome in human islets. NLRP3 inflammasome expression and IL-1β production have been shown to be induced in mouse islet beta cells in response to glucose, even though to a lower level than that observed in macrophages^20^. With regard to human islets, it is already known that glucose leads to IL-1β secretion^21^ but NLRP3 inflammasome expression has not yet been demonstrated in human islets. Here, using different approaches, we showed that NLRP3 inflammasome is expressed in human islet cells. In macrophages, NLRP3 inflammasome expression is induced in response to LPS through its receptor TLR4. Studies have shown that TLR4 is also expressed in human islet cells suggesting that NLRP3 inflammasome could be activated in human islets in response to LPS + ATP^22,23^. In our study, we demonstrated that NLRP3 inflammasome activation in human islets is dependent on the presence of LPS + ATP. Indeed, human islets exposed to LPS + ATP exhibit an increase in *IL1B* gene expression and secretion. By immunofluorescence, IL-1β is shown in beta and non-beta cells and, interestingly, LPS + ATP treatment increases IL-1β staining in beta cells but not in non-beta cells. Furthermore, inhibition of NLRP3 inflammasome by glyburide prevents the effects of LPS + ATP on gene expression, content and secretion of IL-1β and gene expression of *NLRP3*, demonstrating that NLRP3 inflammasome is a regulated process. Our results are in agreement with the study of Tschopp et al., who showed that NLRP3 inflammasome is expressed and inhibited by glyburide in murine islet cells^20^.

NLRP3 inflammasome is involved in metabolic disorders, such as type 2 diabetes and obesity^24,25^. NLRP3-deficient mice display a greater glucose tolerance and insulin sensitivity^20^, a reduction of inflammation in adipose tissue and liver, and a protection against obesity-induced insulin resistance^26^. Moreover, in vitro experiments have shown that saturated fatty acids and IAPP trigger activation of NLRP3 inflammasome leading to production of IL-1β and caspase-1^27,28^.

NLRP3 inflammasome has been also studied in T1D. Genetic studies highlighted an upregulation of TLR4 and TLR2 in patients with T1D^29^ and two polymorphisms in NLRP3 were identified as a predisposing factor for T1D^30^. Interestingly, NLRP3-deficient NOD mice were shown to be protected from T1D development^31^. However, whether local islet production and activation of NLRP3 inflammasome is involved in inflammatory events and/or beta cell destruction in T1D has yet to be investigated. Pancreatic islet transplantation is a cell therapy procedure for T1D. In the pathogenesis of transplant rejection, inflammasome pathways have been shown to be involved in acute and chronic rejection of solid organs^9,32,33^. However, involvement of inflammasome in grafted islets has not yet been studied. We hypothesized that hypoxic conditions, to which islets are exposed during isolation and after transplantation, could lead to NLRP3 inflammasome activation. Indeed, immediately after transplantation and up to 10 days post-transplant, islets are not vascularized and undergo hypoxia. To assess the effect of hypoxia on inflammasome activation, isolated human islets were incubated 24 h in vitro at 1% O_2_ to mimic a severe hypoxia. Our results showed that hypoxia increases gene expression of *NRLP3* and *IL1B* and induces IL-1β and caspase-1 production in human islets, demonstrating that hypoxia is an enhancer of NLRP3 inflammasome. These results correlate with studies describing ROS, generated by hypoxia-induced oxidative stress, as activators of NLRP3 inflammasome^13–15,20^. Zhou et al.^20^ have shown that NLRP3 interacts with thioredoxin (TRX)-interacting protein (TXNIP), leading to inflammasome assembly, in mouse islet cells exposed to oxidative stress. Interestingly, we observed an increase in *TXNIP* gene expression in response to hypoxia in human islets (data not shown) suggesting that activation of NLRP3 inflammasome could be mediated by TXNIP as observed in mouse islet cells^20^. We then studied whether NLRP3 inflammasome is involved in hypoxia-induced damage in islets. As expected, hypoxia induces apoptosis and necrosis in human islets; however glyburide does not prevent hypoxia-induced cell death, indicating that NLRP3 inflammasome is not involved in islet cell death induced by hypoxia. To understand the mechanism involved in hypoxia-induced islet cell death is interesting but out of the scope of this study. In other studies, using different cell systems, mechanisms involved in hypoxia-induced cell death have been described. For instance, activation of intrinsic apoptotic pathway characterized by cytochrome c release, activation of caspase-9 and caspase-3^34,35^ and transcription factors, such as HIF-2, p53, and NF-ĸB, were reported to mediate cell death induction in response to hypoxia^34,36–40^.

Interestingly, a study has shown that activation of NLRP3 inflammasome, induced by high glucose concentrations, endoplasmic reticulum or and oxidative stress, is not involved in mouse islet cell death^41^. It could be possible that the amount of IL-1β, produced by human islets via NLRP3 inflammasome activation, is insufficient to cause islet cell death. IL-1β may associate with others cytokines to induce cell death^42^. Apoptosis has been observed only in human islet cells treated with IL-1β plus interferon-γ, not with IL-1β alone^43^. Furthermore, these cytokines activate p38 involved in β-cell death^44^. In allograft dysfunction, injury and rejection, NLRP3 inflammasome process occurs very early in the inflammatory cascade^9^. In islet graft, production of IL-1β alone could play an immunological role in inducing the recruitment of immune cells, such as dendritic cells and T cells. The activation of these immune cells by IL-1β in islet graft could stimulate the production of inflammatory cytokines such as TNFα and IL-1β leading to dysfunction and destruction of islet graft. Further studies are necessary to understand the role of NLRP3 inflammasome-produced IL-1β in human islet grafts.

## Electronic supplementary material

Figure S1

Figure S2
