# Supplementary figures and images for: NLRP3 inflammasome is expressed and regulated in human islets
Source: Cell Death Dis. 2018 Jun 25;9(7):726. doi: 10.1038/s41419-018-0764-x (PMC6018156; doi:10.1038/s41419-018-0764-x)

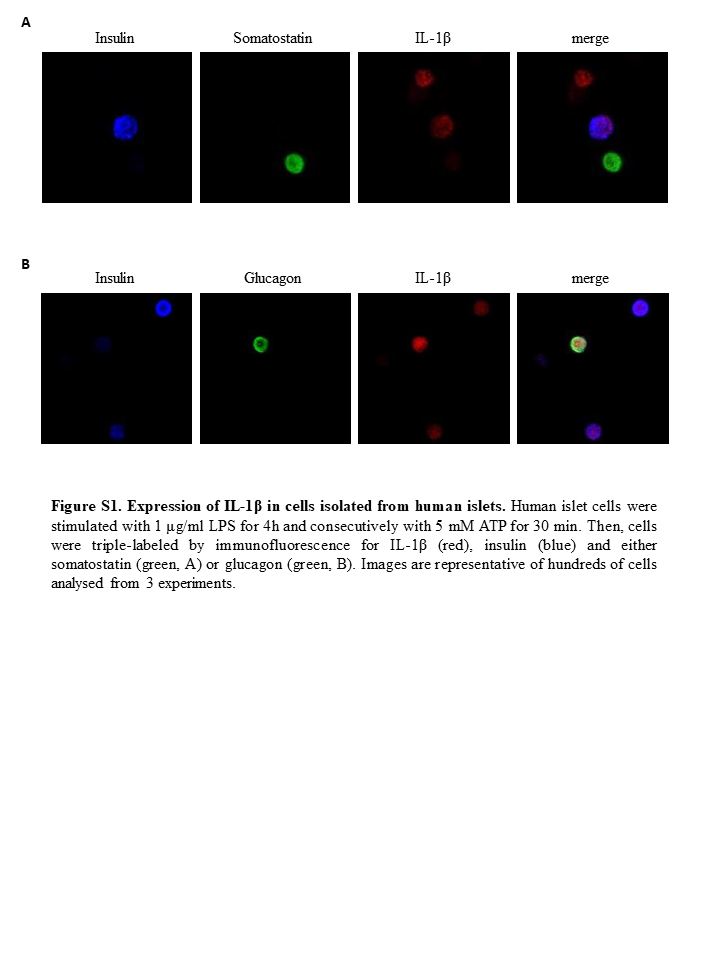

Supplement: Supplementary file 1 — Figure S1 [file 41419_2018_764_MOESM1_ESM.tif]

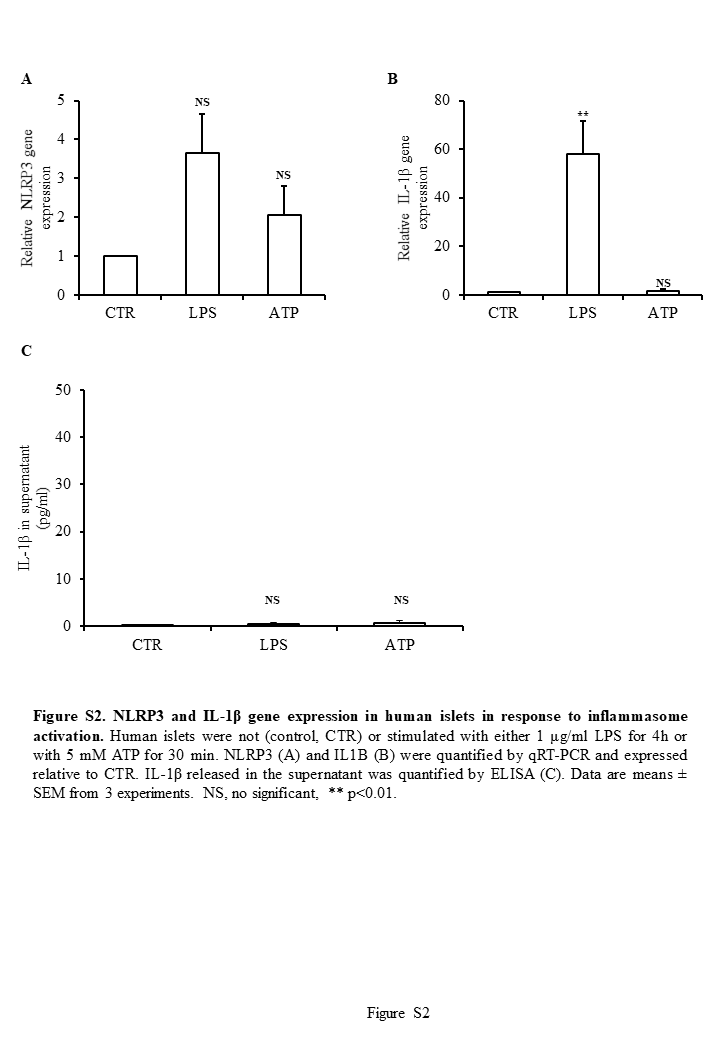

Supplement: Supplementary file 2 — Figure S2 [file 41419_2018_764_MOESM2_ESM.tif]
